# Reviewed article: Bastos MES, Nascimento AJF, Soares MTF, Spinelli MBAS, Low ST. Knowledge, attitudes and practices of pregnant women for preventing risks and accidents with their newborns: cross-sectional study, Recife, 2024. Epidemiol Serv Saude. 2026:35;e20240822

**DOI:** 10.1590/S2237-96222026v35e20240822.b

**Published:** 2025-12-08

**Authors:** Mariana Gaspar Botelho Funari de Faria

**Affiliations:** 1 Universidade de São Paulo, Ribeirão Preto, SP, Brazil

## Peer review B

## Questionnaire

Does the manuscript contain new and significant information to justify publication? Yes

Does the Abstract (Summary) clearly and accurately describe the content of the article? Yes

Is the problem significant and concisely stated? No

Are the methods described comprehensively? Yes

Are the interpretations and conclusions justified by the results? Yes

Is adequate reference made to other work in the field? Yes

## Manuscript Structure

Length of article is: Adequate

Number of tables is: Adequate

Number of figures is: Adequate

Please state any conflict(s) of interest that you have in relation to the review of this paper. None

Interest: Excellent

Quality: Good

Originality: Excellent

Overall: Average

Recommendation: Major Revision

## Comments

O artigo possui algumas fragilidades, porém aborda uma temática importante no que diz respeito à saúde pública e suas contribuições para o conhecimento e manejo adequado do recém nascido por parte das genitoras. As condutas e rotinas de um serviço de promoção à saúde como é o caso das unidades de atenção primaria, produzem um impacto significativo na vida dos pacientes e também dos profissionais ali inseridos. Dessa forma, todos estudos inerentes as práticas assistências e seus desdobramentos possuem grande relevância nacional e internacional.

A linguagem empregada pelos autores está adequada, é coerente e concisa. Entretanto, o termo recém-nascido, é empregado para uma criança nascida e o estudo aborda um contexto de gestantes e seus conhecimentos acerca dos cuidados à serem prestados ao bebe que ainda ira nascer. 

Sugiro utilizar Guidelines para padronizar esse termo e ficar melhor descrito. Sugiro também realizar uma releitura apurada do estudo, devido à apresentar falhas na pontuação e organização das informações (algumas descritas devem estar na introdução porem estão na discussão). Em relação ao conteúdo abordado, sugiro algumas sugestões de aprimoramento e correção do manuscrito.

Avaliação: 

Palavras Chaves: Sugiro retirar a palavra “Estudo transversal”, não há necessidade de ser uma palavra chave pois é uma forma de desenho do estudo e por não constar nem nos descritores Decs e Mesh.

Introdução:

Sempre ao iniciarmos uma introdução se faz necessário abordar aspectos epidemiológicos que embase a discussões e a pertinência dos estudos, bem como, fundamentem o interesse e a ineditude do tema. Sugiro iniciar esse tópico como esses achados.

É necessário trazer toda conceituação do que se vai revelar através do estudo. O estudo traz muitos aspectos que deveriam ser tratado nesse espaço nas discussões. EX: Síndrome da morte súbita, cuidados com RN, momento gestacional.

Boa parte da discussão deveria estar na introdução e não na discussão, sugiro readaptar.

O 2 paragrafo contem uma informação sem nexo desencontrada, sugiro rever: “Desse modo, entende-se que a gravidez é responsável por acarretar a quem gesta sentimentos de dúvida e insegurança na promoção da segurança e do bem-estar do recém-nascido”.

No 5 paragrafo o termo “falta de atenção adequada” faz menção a que grupo? Mães? Profissionais? Deixar claro.

Os profissionais são parte integrante das explanações feitas introdução, no entanto, somente ao final do tópico fala-se sobre, é necessário trazer conceituar

principalmente o cuidado realizado por eles ao longo do texto, uma vez que, é o ponto chave para se obter conhecimento que são os objetivos do trabalho.

Sugiro também uma explanação breve do que se tem produzido na literatura cientifica a respeito do tema. Isso implica na necessidade do estudo, bem como na

pretensão de publicação.

Os autores trazem um termo inexistente na literatura, mesmo na bibliografia citada “acolhimento pré-natal”. Buscar pelo termo exato na literatura.

Metodologia:

Trata-se de um estudo bem fundamentado e com a metodologia bem descrita, permeando o objeto de estudo e descrevendo de maneira objetiva os passos seguidos pelos autores.

Ao se realizar o teste Qui quadrado para variáveis, é necessário observar os pressupostos, os autores não dão informações sobre isso. Sugiro deixar isso claro.

Uma indagação: Qual foi o padrão de comparação entre multíparas e primíparas uma vez que, as multíparas já se depararam com sentimentos e ações em relação aos RNs e as primíparas ainda não conhecem tal fato? São dois grupos distintos, para responderem questionamentos que compilam informações intimas a um evento, que um grupo teve acesso e outro não.

E necessário rever esse questionamento porque os grupos não estão homogêneos para serem classificados de acordo com o Inquérito.

Resultados:

Tópico bem descrito.

Discussão:

Boa parte da introdução encontra-se nesse tópico, sugiro uma leitura rigorosa afim de remanejar as informações de forma coerente.

Os autores trazem como limitação do estudo o quantitativo de gestante, porem fundamentaram na metodologia, um ponto descredibiliza o outro, outro ponto citado, foi de observação por parte profissionais, o objetivo foi a aplicação do inquérito com as gestantes, aonde que os profissionais ajudariam na análise? O papel deles é informativo!

De outra forma não seria relevante esse levantamento.

Rever isso entre os autores.

Referencias:

Pequena parte das referências se encontram além de 5 anos, portanto são validas para amparar as discussões.

